# Supplementary material for: Semi-supervised integration of single-cell transcriptomics data
Source: Nat Commun. 2024 Jan 29;15:872. doi: 10.1038/s41467-024-45240-z (PMC10825117; doi:10.1038/s41467-024-45240-z)
Supplement: Supplementary file 1 — Supplemental Information [file 41467_2024_45240_MOESM1_ESM.pdf]

SUPPLEMENTARY FIGURES

Semi-supervised integration of single-cell transcriptomics data

M. Andreatta et al. (2024)

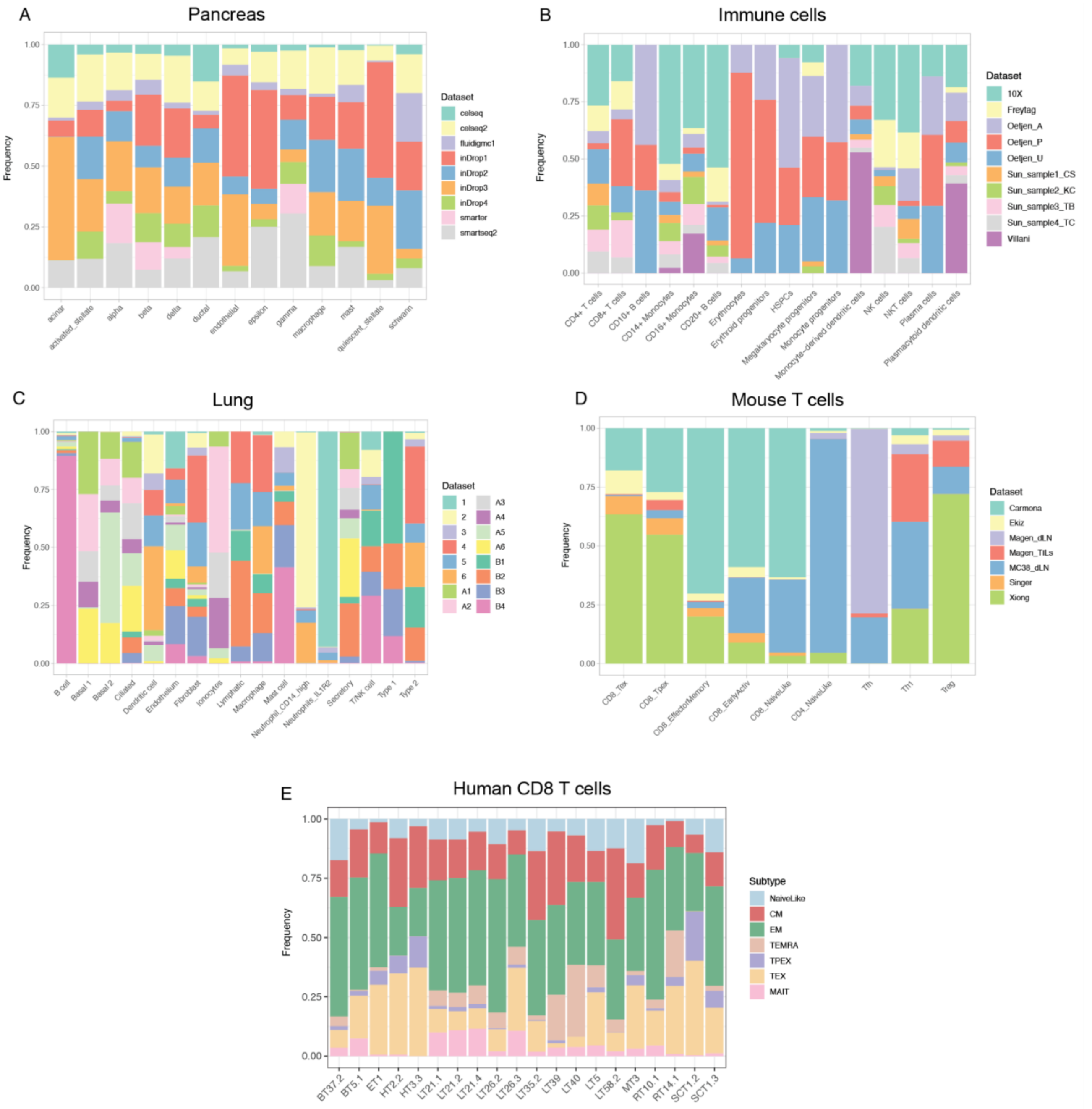

Figure S1: Dataset composition for individual cell types in 4 integration tasks. A) The Pancreas dataset is “balanced” in terms of cell type composition, with most cell types represented in the majority of datasets. **B)** In the Immune cell dataset several cell types are only represented in 3 of the 10 datasets. **C)** The lung integration task is challenging because it comprises samples from multiple human donors, covering different spatial locations, resulting in an imbalanced composition. **D)** The T cell dataset has also high cell type imbalance, with several cell type being represented by only one or few datasets. **E)** Cell type composition of the 20 samples used to construct the human CD8T map. Source data are provided as a Source Data file.

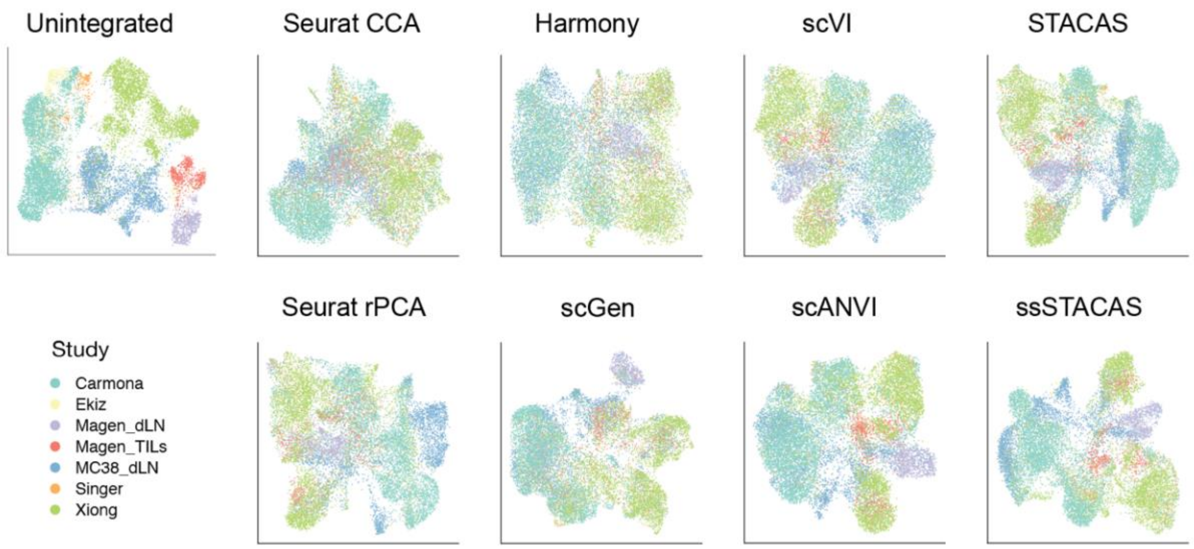

Figure S2: UMAP embeddings for the mouse T cell integration task colored by batch, before integration (Unintegrated) and after integration using eight different integration methods.

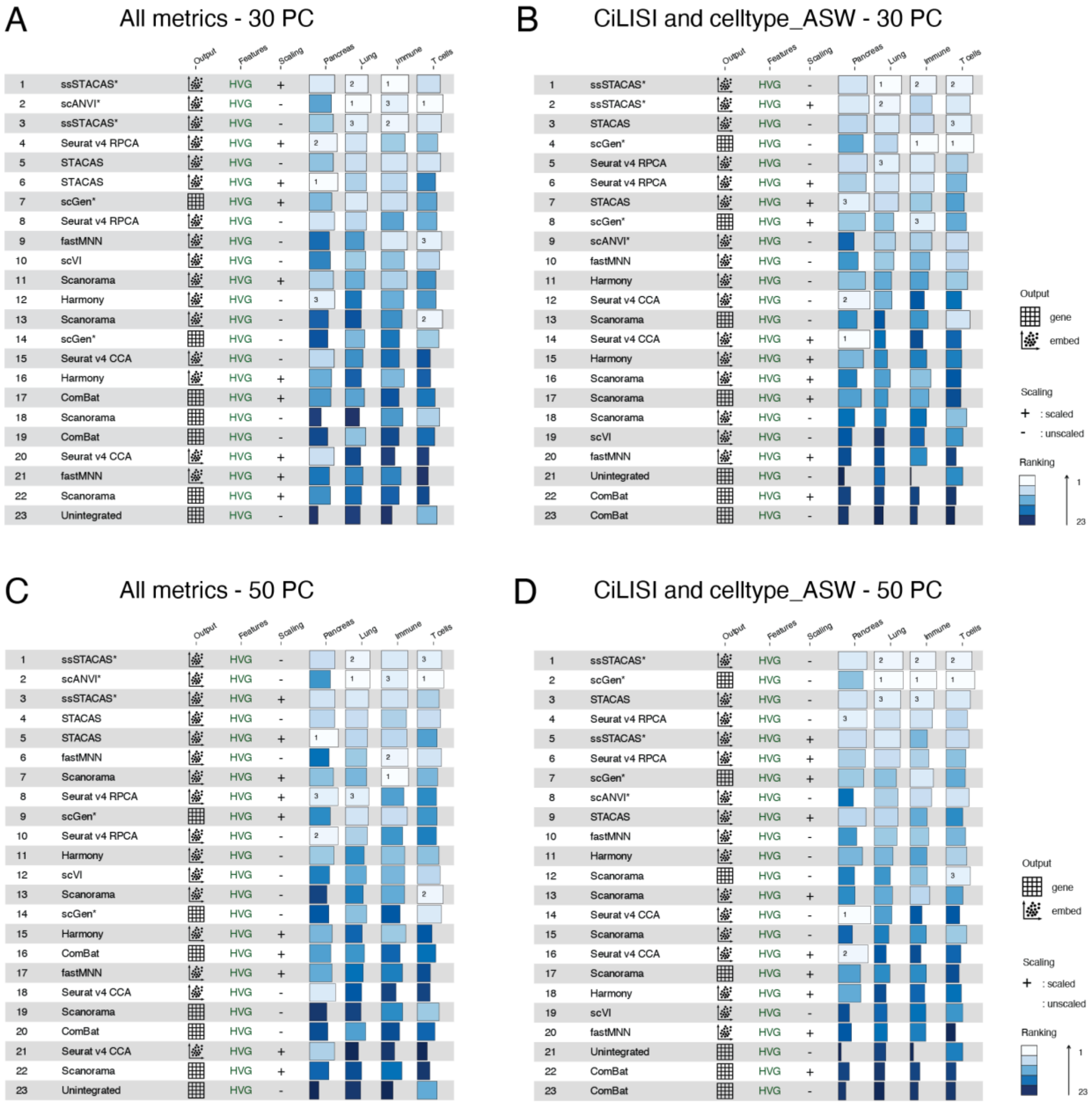

Figure S3: Global rankings for method performance using different metrics and dimensionality reduction sizes. A) Global rankings of integration tools based on the weighted contribution of multiple metrics proposed by Luecken et al., using 30-dimensional latent space (e.g. number of principal components for dimensionality reduction). **B)** Global rankings of integration tools based on the weighted contribution of CiLISI (quantifying batch mixing) and celltype_ASW (quantifying preservation of biological variance), and 30-dimensions latent space. **C)** As A) but using 50-dimensions latent space. **D)** As B) but using 50-dimensions latent space. Source data are provided as a Source Data file.

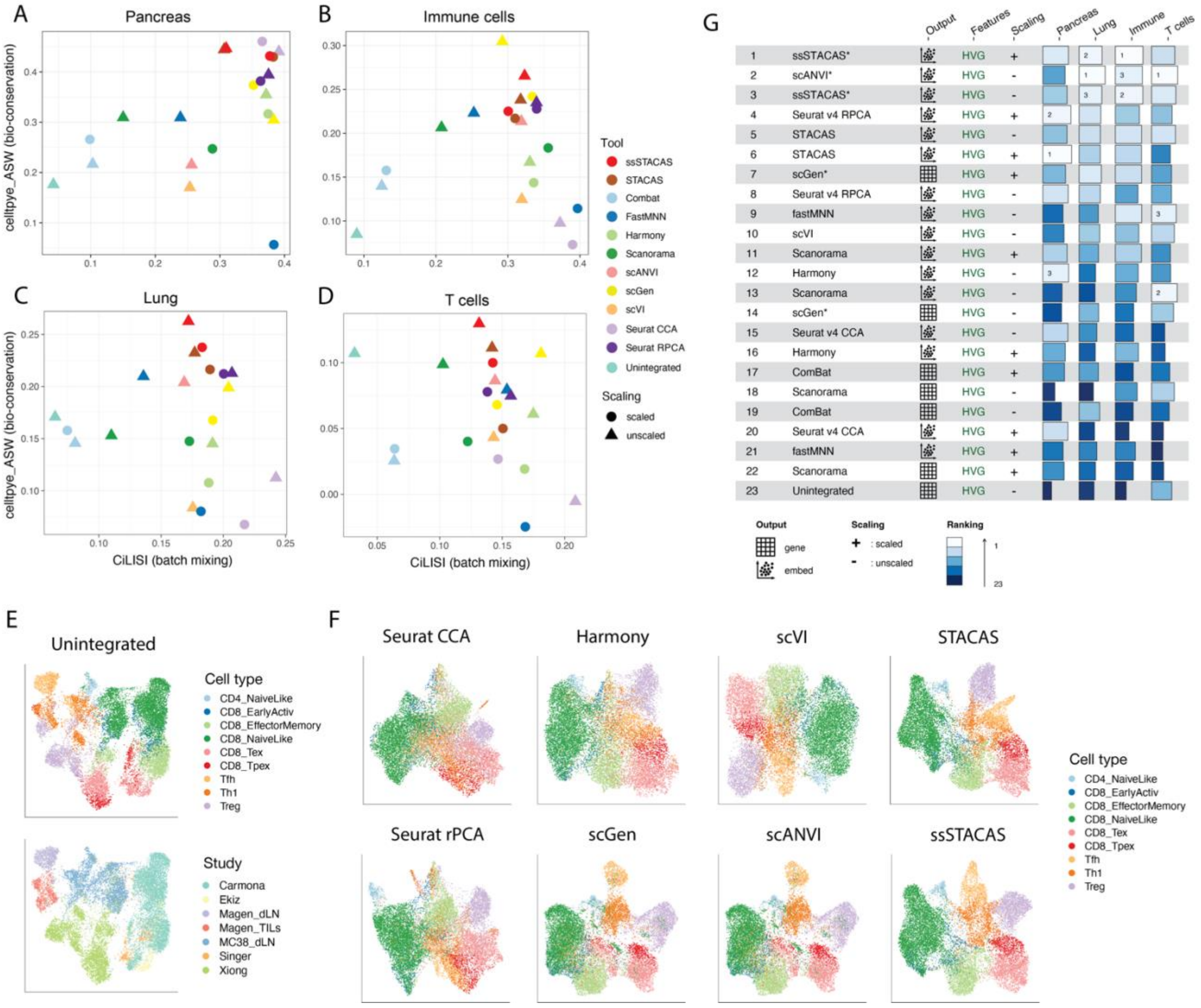

Figure S4: Integration performance for single-cell data integration tools over 4 different tasks, using a 30-dimensional latent space. A) CiLISI (per cell type integration LISI, measuring cell type-aware batch mixing) vs. celltype_ASW (cell type average silhouette width, measuring preservation of biological variance) for several integration methods on the Pancreas integration task. **B)** CiLISI vs. celltype_ASW across methods on the Lung integration task. **C)** CiLISI vs. celltype_ASW across methods on the Immune cells integration task. **D)** CiLISI vs. celltype_ASW across methods on the T cells integration task. **E-F)** UMAP embeddings for the mouse T cell integration task, for unintegrated data colored by cell type (top) and by study of origin (bottom) **(E)** and for eight representative integration methods, colored by cell type **(F)**. **G)** Global rankings of integration tools based on the weighted contribution of a broad panel of metrics both for preservation of biological variance ("bio-conservation") and batch-correction, as proposed by Luecken *et al.* Source data are provided as a Source Data file.

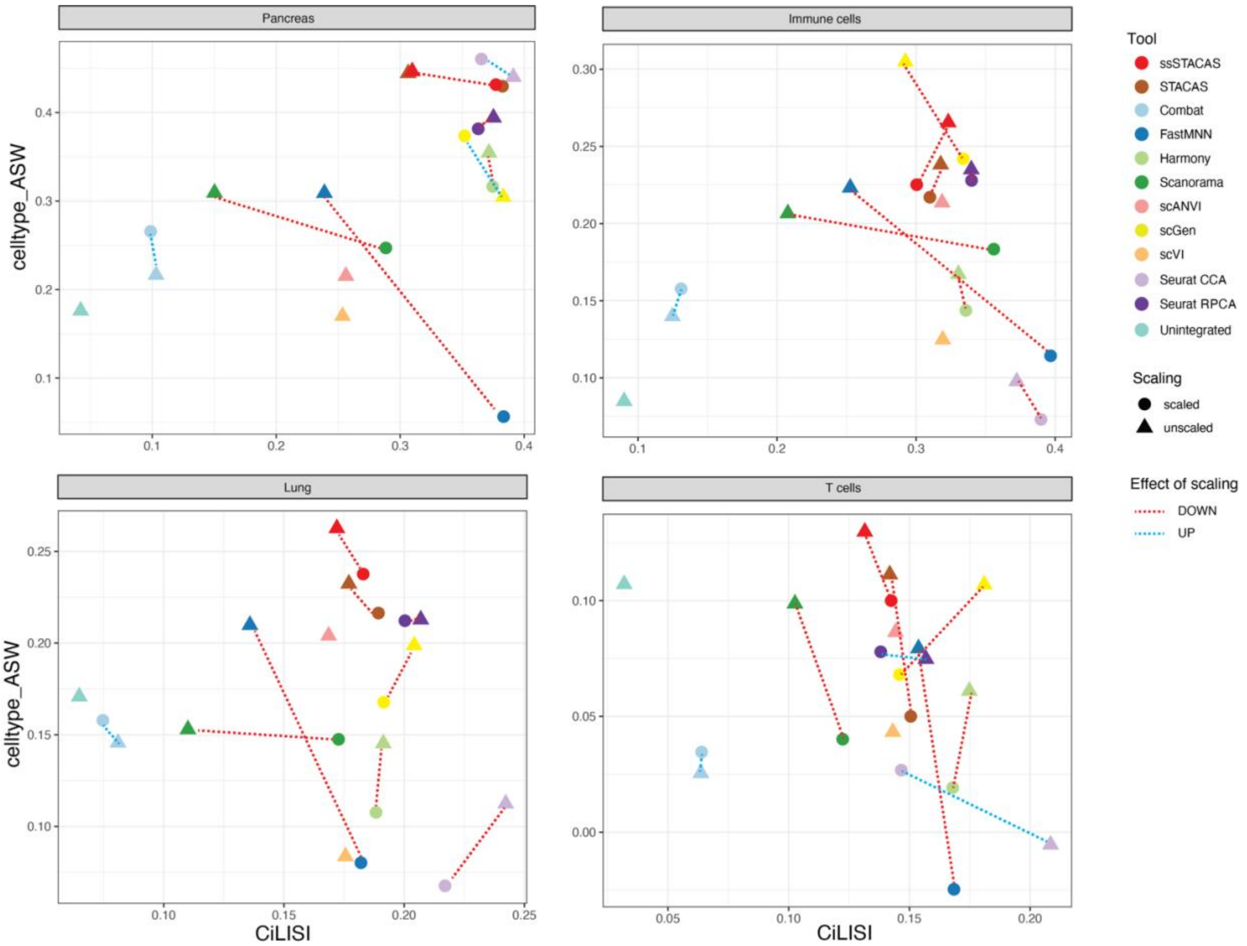

Figure S5: Effect of scaling on integration performance. CiLISI (batch mixing) versus celltype_ASW (preservation of biological variance) for indicated computational tools over 4 integration tasks. Dashed lines connect performance coordinates for the same tool with or without prior data scaling; blue lines indicate tools for which scaling increased celltype_ASW, red lines indicate tools for which scaling had negative effect on celltype_ASW. Source data are provided as a Source Data file.

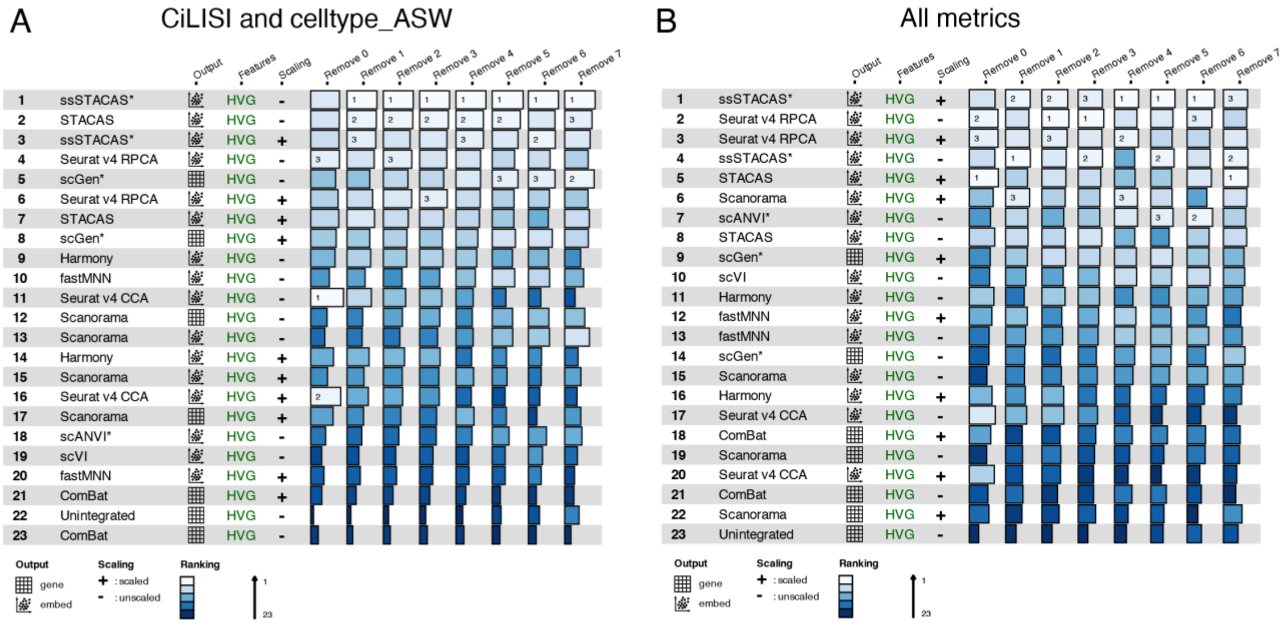

Figure S6: Global ranking of integration tools on Pancreas dataset with increasing levels of cell type imbalance. Datasets 'Remove N' (with N between 0 and 7) were generated by randomly removing N cell types from each sample. The plots display combined integration scores calculated using the 'scib' pipeline, and 1,2,3 identify the top three ranking methods for each integration task. **A)** Methods ranking based on CiLISI and celltype_ASW metrics; **B)** Methods ranking based on combination of multiple integration metrics from the 'scib' pipeline. Source data are provided as a Source Data file.

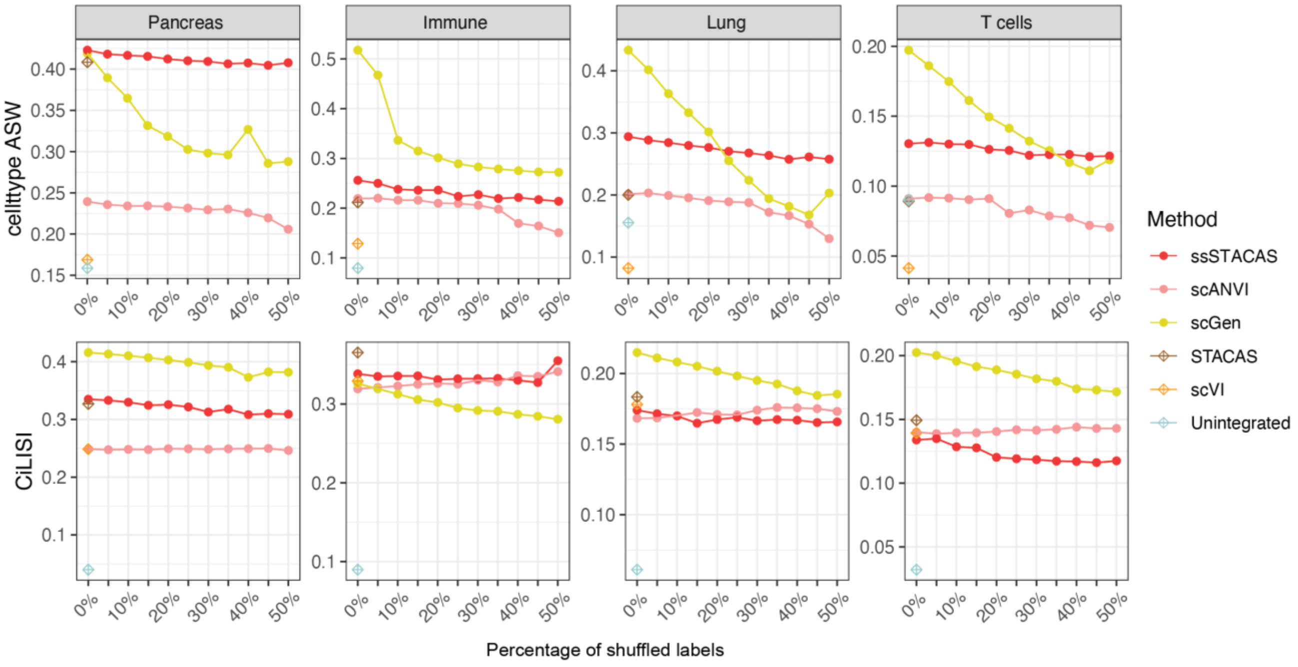

Figure S7: Effect of noisy cell type annotations (by nearest neighbor) on data integration by supervised or semi-supervised methods. Preservation of biological variance (measured by celltype_ASW) and batch mixing (measured by CiLISI) for 4 data integration tasks, using as input all correct cell type labels (0%) or increasing levels of shuffled cell type labels (5% to 50%). In these experiments, labels were shuffled between neighboring cell types, i.e. pairs of cell types with the most similar average expression profiles. Note that shuffling is bounded to 50% of the labels, as higher values would start increasing the probability of cells of the same type being assigned to the same label. Unsupervised versions of ssSTACAS and scANVI (STACAS and scVI respectively) are included for reference. Source data are provided as a Source Data file.

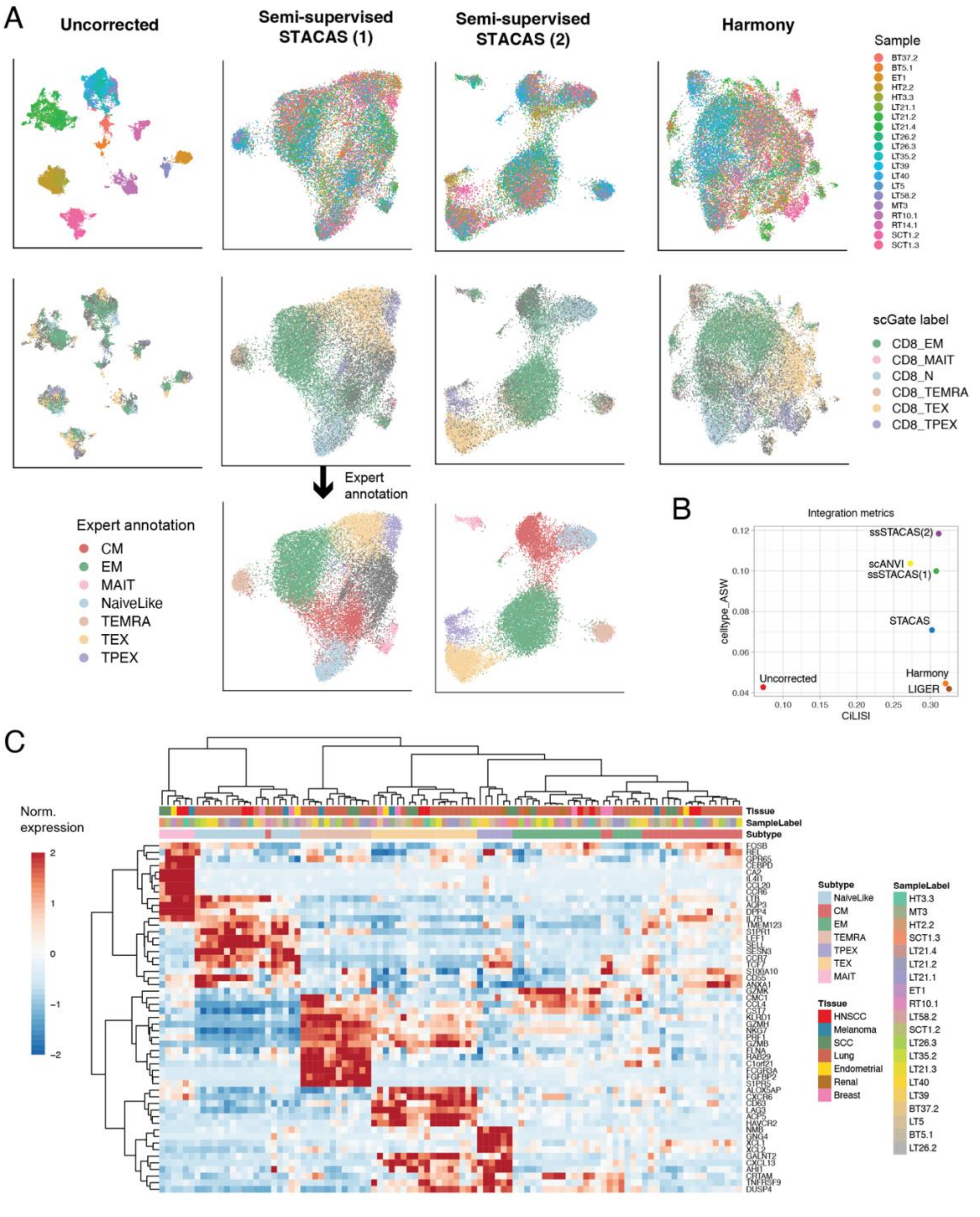

Figure S8: Construction of a reference map for CD8 T cells using semi-supervised STACAS. A) UMAP embeddings for unintegrated data, semi-supervised STACAS guided by scGate predicted cell types (1), semi-supervised STACAS guided by updated labels after expert annotation (2), and the Harmony integration tool. Cells are colored by sample of origin (first row), scGate predicted annotation (second row) and by expert annotation (third row). **B)** Batch mixing (measured by CiLISI) and bio-conservation (measured by celltype_ASW) for the indicated algorithms on the collection of CD8 T cell samples. **C)** Average normalized RNA expression for differentially expressed genes in individual subtype-sample combinations of the CD8 T reference map. For all subtypes with at least 30 cells in a sample, the average normalized RNA expression was centered and rescaled by the standard deviation by gene. Profiles are shown for genes that were differentially expressed (log-fold change > 0.5) consistently in least 80% of the datasets. Hierarchical clustering using the Ward D2 algorithm shows that expression profiles are grouped largely by T cell subtype rather than by study or tissue of origin. Source data are provided as a Source Data file.

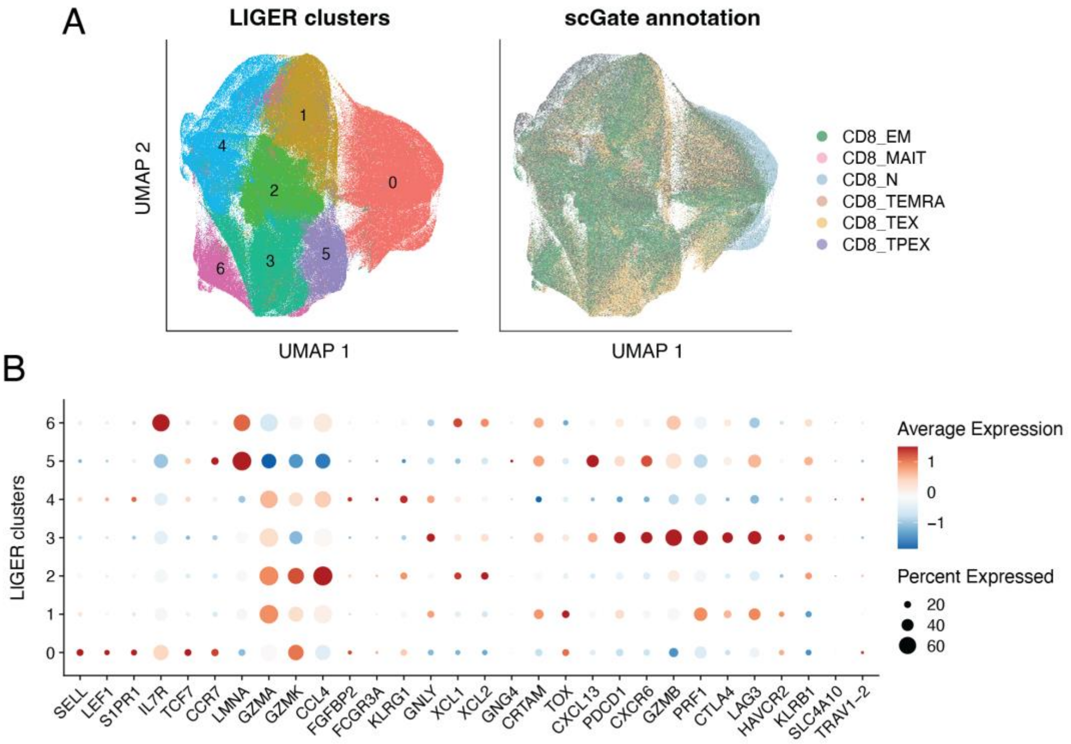

Figure S9: Large scale integration of CD8 T cells using LIGER. A) UMAP embeddings of 285 samples integrated using LIGER in online-learning mode, colored by unsupervised cluster (left) and by predicted scGate CD8 T cell subtypes. **B)** Expression profiles for unsupervised clusters obtained by online LIGER integration, using a panel of standard marker genes for human CD8 T cell subtypes. Source data are provided as a Source Data file.
