# Supplementary material for: Semi-supervised integration of single-cell transcriptomics data
Source: Nat Commun. 2024 Jan 29;15:872. doi: 10.1038/s41467-024-45240-z (PMC10825117; doi:10.1038/s41467-024-45240-z)
Supplement: Supplementary file 3 — Reporting Summary [file 41467_2024_45240_MOESM3_ESM.pdf]

nature portfolio

Corresponding author(s): Santiago Carmona
Last updated by author(s): 18/12/23

Reporting Summary

Nature Portfolio wishes to improve the reproducibility of the work that we publish. This form provides structure for consistency and transparency in reporting. For further information on Nature Portfolio policies, see our Editorial Policies and the Editorial Policy Checklist.

Statistics

For all statistical analyses, confirm that the following items are present in the figure legend, table legend, main text, or Methods section.

n/a	Confirmed	
[ ]	[x]	The exact sample size (*n*) for each experimental group/condition, given as a discrete number and unit of measurement
[x]	[ ]	A statement on whether measurements were taken from distinct samples or whether the same sample was measured repeatedly
[x]	[ ]	The statistical test(s) used AND whether they are one- or two-sided<br>*Only common tests should be described solely by name; describe more complex techniques in the Methods section.*
[ ]	[x]	A description of all covariates tested
[ ]	[x]	A description of any assumptions or corrections, such as tests of normality and adjustment for multiple comparisons
[ ]	[x]	A full description of the statistical parameters including central tendency (e.g. means) or other basic estimates (e.g. regression coefficient) AND variation (e.g. standard deviation) or associated estimates of uncertainty (e.g. confidence intervals)
[x]	[ ]	For null hypothesis testing, the test statistic (e.g. *F*, *t*, *r*) with confidence intervals, effect sizes, degrees of freedom and *P* value noted<br>*Give P values as exact values whenever suitable.*
[x]	[ ]	For Bayesian analysis, information on the choice of priors and Markov chain Monte Carlo settings
[x]	[ ]	For hierarchical and complex designs, identification of the appropriate level for tests and full reporting of outcomes
[x]	[ ]	Estimates of effect sizes (e.g. Cohen's *d*, Pearson's *r*), indicating how they were calculated

Our web collection on statistics for biologists contains articles on many of the points above.

Software and code

Policy information about availability of computer code

Data collection	No software was used for data collection
Data analysis	STACAS is available as a R package at https://zenodo.org/doi/10.5281/zenodo.10402054 and https://github.com/carmonalab/STACAS<br>The implementation of the performance metrics used in this work can be installed as a package from the following repository: https://github.com/carmonalab/scIntegrationMetrics also available at https://zenodo.org/doi/10.5281/zenodo.10402131<br>The snakemake pipeline that reproduces the results of our benchmark, based on the 'scib' pipeline by Luecken et al., is available at https://zenodo.org/doi/10.5281/zenodo.10402023 and https://github.com/carmonalab/scib-pipeline<br>The code to construct, annotate and use the CD8 T cell reference map is available at: https://github.com/carmonalab/CD8_human_TIL_atlas_construction

For manuscripts utilizing custom algorithms or software that are central to the research but not yet described in published literature, software must be made available to editors and reviewers. We strongly encourage code deposition in a community repository (e.g. GitHub). See the Nature Portfolio guidelines for submitting code & software for further information.

Data

Policy information about availability of data

All manuscripts must include a data availability statement. This statement should provide the following information, where applicable:

- Accession codes, unique identifiers, or web links for publicly available datasets
- A description of any restrictions on data availability
- For clinical datasets or third party data, please ensure that the statement adheres to our policy

For benchmarking, the human pancreas atlas, the human immune cell atlas and the human lung atlas, collated by Luecken et al., were downloaded from figshare (https://doi.org/10.6084/m9.figshare.12420968.v8). The mouse T cell atlas by Andreatta et al. is available from figshare (https://doi.org/10.6084/m9.figshare.12478571).
The T cell processed scRNA-seq dataset collection ("uTILity") used in this study was downloaded from https://doi.org/10.5281/zenodo.6325603. References to the original studies and associated metadata are also available at https://github.com/ncborcherding/utility.

The integrated CD8 T cell reference map is available as a Seurat object at https://doi.org/10.6084/m9.figshare.23608308

Research involving human participants, their data, or biological material

Policy information about studies with human participants or human data. See also policy information about sex, gender (identity/presentation), and sexual orientation and race, ethnicity and racism.

Reporting on sex and gender	n/a
Reporting on race, ethnicity, or other socially relevant groupings	n/a
Population characteristics	n/a
Recruitment	n/a
Ethics oversight	n/a

Note that full information on the approval of the study protocol must also be provided in the manuscript.

Field-specific reporting

Please select the one below that is the best fit for your research. If you are not sure, read the appropriate sections before making your selection.

- [x] Life sciences
- [ ] Behavioural & social sciences
- [ ] Ecological, evolutionary & environmental sciences

For a reference copy of the document with all sections, see nature.com/documents/nr-reporting-summary-flat.pdf

Life sciences study design

All studies must disclose on these points even when the disclosure is negative.

Sample size	In the benchmark we used 4 collections of published, expert annotated datasets ranging from 7 to 16 samples each. These are datasets with sufficient size and biological and technical diversity to evaluate batch effects correction, in line with the current practices and standards in the field.
Data exclusions	No data were excluded from the analyses
Replication	The results showed can be reproduced using the code and data provided
Randomization	The experiments were not randomized
Blinding	The Investigators were not blinded to allocation during experiments and outcome assessment.

Reporting for specific materials, systems and methods

We require information from authors about some types of materials, experimental systems and methods used in many studies. Here, indicate whether each material, system or method listed is relevant to your study. If you are not sure if a list item applies to your research, read the appropriate section before selecting a response.

Materials & experimental systems

n/a	Involved in the study
☒	☐ Antibodies
☒	☐ Eukaryotic cell lines
☒	☐ Palaeontology and archaeology
☒	☐ Animals and other organisms
☒	☐ Clinical data
☒	☐ Dual use research of concern
☒	☐ Plants

Methods

n/a	Involved in the study
☒	☐ ChIP-seq
☒	☐ Flow cytometry
☒	☐ MRI-based neuroimaging
